# Fatty acid metabolites of *Dendrobium nobile* were positively correlated with representative endophytic fungi at altitude

**DOI:** 10.3389/fmicb.2023.1128956

**Published:** 2023-04-27

**Authors:** Yongxia Zhao, Lin Qin, Daopeng Tan, Di Wu, Xingdong Wu, Qingjie Fan, Chaojun Bai, Jiyong Yang, Jian Xie, Yuqi He

**Affiliations:** ^1^Guizhou Engineering Research Center of Industrial Key-technology for Dendrobium Nobile and Engineering Research Center of Pharmaceutical Orchid Plant Breeding and High Efficiency Application in Guizhou Province, Zunyi Medical University, Zunyi, China; ^2^Key Laboratory of Basic Pharmacology of Ministry of Education and Joint International Research Laboratory of Ethnomedicine of Ministry of Education, Zunyi Medical University, Zunyi, China; ^3^2011 Cooperative Inovational Center for Guizhou Traditional Chinese Medicine and Ethnic Medicine Zunyi Medical University, Zunyi, China; ^4^Guangxi Shenli Pharmaceutical Co., Ltd, Yulin, China; ^5^Chishui Xintian Chinese Medicine Industry Development Co., Ltd, Zunyi, China

**Keywords:** *Dendrobium nobile*, altitude, endophytic fungi, fatty acids, metabolites

## Abstract

**Introduction:**

Altitude, as a comprehensive ecological factor, regulates the growth and development of plants and microbial distribution. *Dendrobium nobile (D. nobile)* planted in habitats at different elevations in Chishui city, also shows metabolic differences and endophytes diversity. What is the triangular relationship between altitude, endophytes, and metabolites?

**Methods:**

In this study, the diversity and species of endophytic fungi were tested by ITS sequencing and metabolic differences in plants were tested by UPLC–ESI–MS/MS. Elevation regulated the colonization of plant endophytic fungal species and fatty acid metabolites in *D. nobile*.

**Results:**

The results indicate that and high altitude was better for the accumulation of fatty acid metabolites. Therefore, the high-altitude characteristic endophytic floras were screened, and the correlation with fatty acid metabolites of plants was built. The colonization of *T. rubrigenum, P. Incertae sedis unclassified, Phoma. cf. nebulosa* JZG 2008 and Basidiomycota unclassified showed a significantly positive correlation with fatty acid metabolites, especially 18-carbon-chain fatty acids, such as (6Z,9Z,12Z)-octadeca-6,9,12-trienoic acid, 3,7,11,15-tetramethyl-12-oxohexadeca-2,4-dienoic acid and Octadec-9-en-12-ynoic acid. What is more fascinating is these fatty acids are the essential substrates of plant hormones.

**Discussion:**

Consequently, it was speculated that the *D. nobile*- colonizing endophytic fungi stimulated or upregulated the synthesis of fatty acid metabolites and even some plant hormones, thus affecting the metabolism and development of *D. nobile*.

## Introduction

*Dendrobium nobile*, a Chinese medicinal material with a long history, is immunomodulator, antitumor agent, antidiabetic (Teixeira and Ng, 2017), antioxidant (Hsu et al., 2022), potential Alzheimer treatment agent (Li D. D. et al., 2022), protection agent of the liver (Zhou et al., 2020), and even the protection agent of retina (Hsu et al., 2022), with its stems as the main medicinal part. A variety of metabolites of *D. nobile*, including alkaloids, lipids, phenolic acids, flavonoids, polysaccharides, and other bioactive substances (Lu et al., 2022), provide a chemical basis for pharmacological action. The synthesis and accumulation of these chemicals are affected by the biological and abiotic environment of the planting habitats (Mykhailenko et al., 2020). At present, the wild resources of *D. nobile* are very scarce, and Chishui City of Guizhou Province has developed into a top-ranking imitation wild planting base of *D. nobile* in China. However, differences in the chemical composition content in *D. nobile* were found among the different geographical locations of Chishui city. In addition to temperature, light, water and soil fertility, altitude and microbial ecology are also important influencing factors of plant metabolic products (McCormick et al., 2018). Chishui city, which located in the northwest of Guizhou Province, is the transitional zone featuring the special Danxia landform from the Guizhou Plateau to the Sichuan Basin. The Danxia landform was shaped from an erosive landscape developed on red beds, which is characterized by scarp slopes. Therefore, the height of the terrain directly affects the status of Danxia stone (red beds). *D. nobile* is the lovely scenery growing on Danxia Stone. Due to the existence of iron oxides, the red beds are mainly red, and iron oxides are sedimentary rocks, usually consisting of conglomerate, glutenite, sandstone, siltstone, shale, and mudstone (Yan et al., 2019). These types of rocks are soft and rich in nutrients, so a large number of moss and microorganisms adhered to danxia stone, which also provides favorable ecology for the growth of *D. nobile*. Accordingly, habitats at different elevations as well as different microbial biological conditions were conjectured to be the important factors for the accumulation of plant metabolites of *D. nobile*.

Medicinal plants will coevolve with their habitats through natural selection at high altitudes, and their chemical and pharmacological effects will change significantly compared with those at low altitudes. Massive healthy plants are colonized by rich and diverse microbial communities, which can regulate the physiology and development of plants. Similarly, the diversity of the microbial population colonizing plants is closely related to external environmental factors. Besides providing nutrition for plants, plant metabolites also play many other roles, including defense against pathogens, pests and herbivores. Moreover, it can be employed as environmental stress, mediate interactions between organisms. Unquestionable, plant endophytes participate in many of these processes directly or indirectly by regulating plant metabolism (Pang et al., 2021). After initially invading plants, microorganisms release effects to evade or hinder the immune recognition of plants and then finally execute plant defense through signal transduction. Phytohormone signaling is one of the most important signal transduction processes in plants (Wang et al., 2022). Furthermore, most plant hormones are synthesized by fatty acid synthesis. Therefore, fatty acids (FAs) can regulate signal transduction as a result of environmental and developmental stimuli and thus play an important role in the composition and fluidity of plant cell membranes and intracellular signal transduction affecting the plant immune response (Walley et al., 2013). Further, the crosstalk interaction triangle between external environmental factors--plant endophytes--plant metabolites was revealed. Consequently, the status of fatty acids is particularly important in exploring the relationship between plant endophytes and host metabolism. Currently, the root colonization of arbuscular mycorrhizal, ectomycorrhizal and mycorrhizal fungi plays an important role in global plant evolution, distribution and adaptability. In contrast, the physiological correlation of fungal group members who do not establish symbiotic structures but are capable of settling asymptomatic plants in nature is still unclear. Therefore, this paper was committed to discovering the triangular relationship between the diversity and abundance of endophytes, environmental factors and host metabolites.

## Materials and methods

### Sample collection

The 24 samples of *D. nobile* stem grown on Danxia stone were obtained from the 327 m, 484 m, 528 m and 692 m of the imitation wild planting base in Chishui city, Guizhou (N28°30′2″, E105°55′48″), and each altitude contained six biological replicates. After removing the roots and leaves, the stems were divided into two parts: one part with 200 mg of each sample was disinfected as follows. First, 20 ml 75% alcohol was added to a 50 ml tube for 45 s. Second, after alcohol removal, 20 ml 0.1% mercuric chloride was added to deepen disinfection for 5 min. Finally, sterile water was used to rinse the remaining mercuric chloride three times. All sterile stems were immediately stored at −80°C and then sent to the Lianchuan Biological Company for internal transcribed spacer (ITS) sequencing. The other clean fresh *D. nobile* stems were dried at 60°C in a drying oven. The dried stems were ground into fine powder (300 mesh), then 75 mg was soaked in 1 ml of 70% (v/v) methanol, followed by ultrasonication (50 kHz, 400 W) for 30 min. Next, the liquid was centrifuged at 9705 g for 5 min, and the supernatant was used for UPLC–ESI–MS/MS analysis.

### Total DNA extraction and high-throughput sequencing of *Dendrobium nobile*

To characterize the endophytic fungal community in *D. nobile* at different altitudes, genomic DNA from all stems was extracted using the E.Z.N.A. Stool DNA Kit (D4015, Omega, Inc., United States). Approximately 200 mg fresh stems were ground and transferred into SLX-Mlus buffer (540 μL). Then, 60 μL of DS buffer and 20 μL of proteinase K solution were added, the mixture was incubated at 70°C for 10 min. Later, the supernatant of the mixture was collected for purification after SP2 buffer was added. Finally, the total DNA was eluted in a tube with 50 μL of elution buffer and stored at −80°C until PCR was performed at LC-Bio Technology Co., Ltd., Hangzhou, Zhejiang Province, China. The ITS2 region of the fungi was amplified with slightly modified versions of the primers ITS1FI2 (5′-GTGARTCATCGAATCTTTG-3′) and ITS2 (5′-TCCTCCGCTTATTGATATGC-3′). Amplification, purification and sequencing library construction were performed as described in (Li L. et al., 2022). The libraries were sequenced on the NovaSeq PE250 platform.

### Sample preparation and extraction for UPLC–ESI–MS/MS analysis

Biological samples were freeze-dried by a vacuum freeze-dryer (Scientz-100F). The freeze-dried sample was crushed using a mixer mill (MM 400, Retsch) with zirconia beads for 1.5 min at 30 Hz. Fifty milligrams of lyophilized powder were dissolved in 1.2 ml 70% methanol solution and vortexed for 30 s every 30 min for a total of 6 times. Following centrifugation at 12,000 rpm for 3 min, the extracts were filtered (SCAA-104, 0.22 μm pore size; ANPEL, Shanghai, China)^1^ before UPLC–MS/MS analysis.

### UPLC conditions

The sample extracts were analyzed using a UPLC–ESI–MS/MS system (UPLC, SHIMADZU Nexera X2^2^; MS, Applied Biosystems 4,500 Q TRAP^3^). The analytical conditions were as follows: UPLC: column, Agilent SB-C18 (1.8 μm, 2.1 mm * 100 mm). The mobile phase consisted of solvent A, pure water with 0.1% formic acid, and solvent B, acetonitrile with 0.1% formic acid. Sample measurements were performed with a gradient program that employed the starting conditions of 95% A and 5% B. Within 9 min, a linear gradient to 5% A and 95% B was programmed, and a composition of 5% A and 95% B was maintained for 1 min. Subsequently, a composition of 95% A and 5.0% B was adjusted within 1.1 min and maintained for 2.9 min. The flow velocity was set as 0.35 ml per minute. The column oven was set to 40°C, and the injection volume was 4 μL. The effluent was alternatively connected to an ESI-triple quadrupole-linear ion trap (QTRAP)-MS.

### ESI-q trap-MS/MS

The ESI source operation parameters were as follows: source temperature 550°C; ion spray voltage (IS) 5,500 V (positive ion mode)/−4,500 V (negative ion mode); ion source gas I (GSI), gas II (GSII), and curtain gas (CUR) were set at 50, 60, and 25 psi, respectively; and collision-activated dissociation (CAD) was high. Instrument tuning and mass calibration were performed with 10 and 100 μmol/l polypropylene glycol solutions in QQQ and LIT modes, respectively. QQQ scans were acquired as MRM experiments with collision gas (nitrogen) set to medium. The declustering potential (DP) and collision energy (CE) for individual MRM transitions were determined with further DP and CE optimization. A specific set of MRM transitions was monitored for each period according to the metabolites eluted within this period.

### Statistical analysis of endophytic fungi

The four samples were sequenced on the Illumina NovaSeq platform. After removal of the primer adapter sequence, the raw reads were sorted according to barcode sequences. Paired-end reads were merged using Pear. According to fqtrim software, quality filtering on the raw reads was performed to obtain high-quality clean tags. Vsearch software was used to filter the chimeric sequences. After dereplication by DADA2, the feature table and feature sequences were obtained. OTU Venn diagrams, alpha diversity and beta diversity were calculated in Origin 2021 software. LEfSe analyses were calculated by galaxy.^4^

### Statistical analysis of metabolites

After normalization, the chemometric analyses were encompassed by MetaboAnalyst 5.0,^5^ where they were subjected to ANOVA, hierarchical cluster analysis, correlation analysis, PCA and PLS-DA. Cross-validation and permutation tests were used to evaluate the robustness of the model. The VI*p* value of each variable in the PLS-DA model was calculated to indicate its contribution to the classification. ANOVA was applied to determine the significance of differences among all groups of independent samples. The criteria to screen significantly changed metabolites were both VIP > 1.5 and *p* value <0.05.

### Correlation analysis

Spearman correlation analysis using Origin 2021 software was performed to evaluate the correlation between differential metabolites and predominant endophytes in *D. nobile* from four altitudes. The Spearman correlation coefficient and two-tailed test were used to describe the degree of correlation.

## Results

### The diversity of endophytic fungi and metabolites of *Dendrobium nobile* was not affected by different altitudes

**Figure 1:**
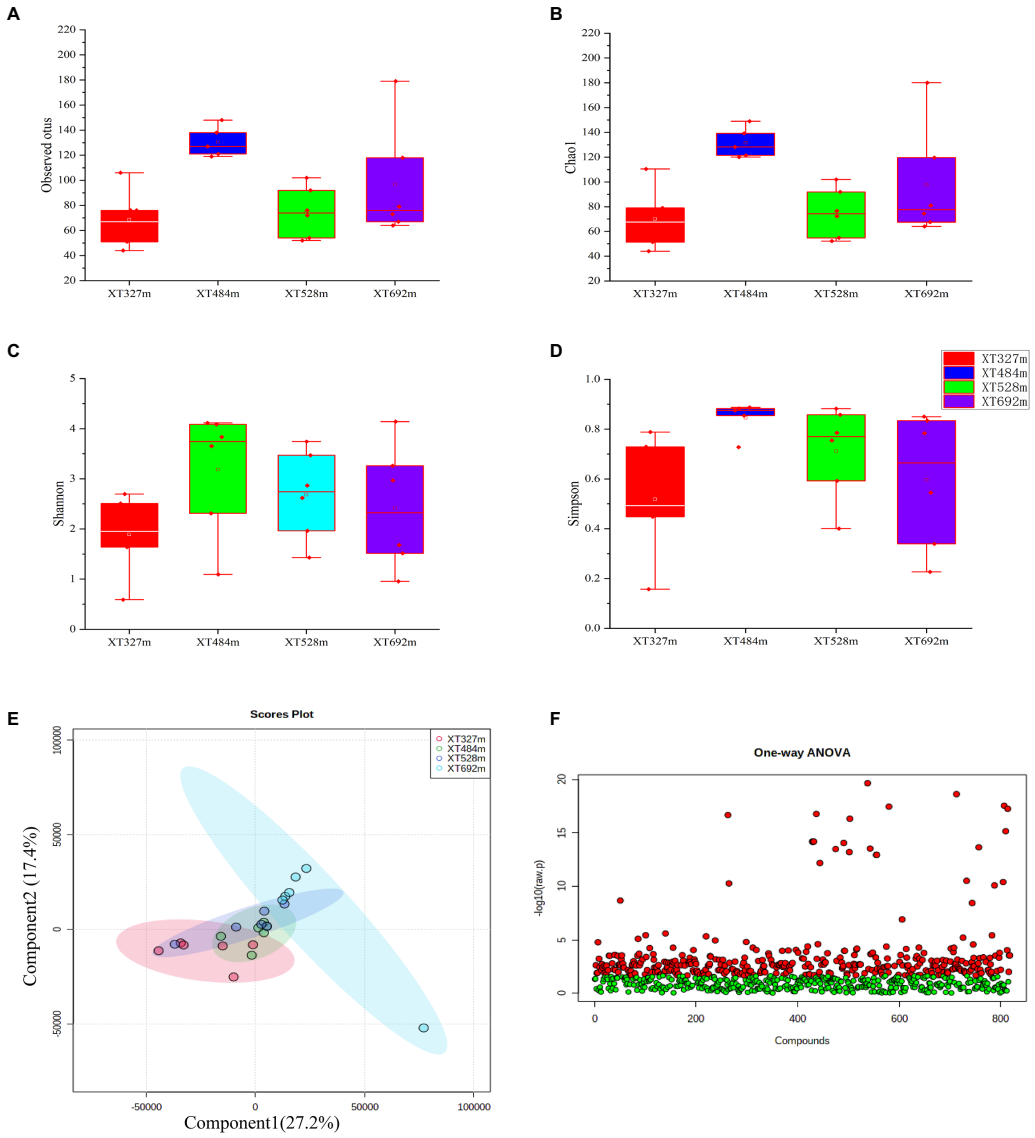
Diversity of endophytic fungi and metabolites in *Dendrobium nobile* (*D. nobile*) at different altitudes. **(A)** The observed OTUs of endophytic fungi in Dendrobium nobile at different altitudes; **(B)** The Chao 1 index of the endophytic fungi and Tukey’s test showed no significant difference (*p* > 0.05), indicating that the number of species in Dendrobium stems is the same at different altitudes. **(C)** The Shannon index of endophytic fungi. **(D)** The Simpson diversity index showed no significant difference between them (*p* > 0.05). **(E)** The β diversity PCA diagram. **(F)** One-way ANOVA of the metabolites in *D. nobile* at different altitudes. A total of 818 metabolites were obtained and identified by wide targeted metabolomic sequencing. Through one-way ANOVA, 325 compounds with significant differences and 493 compounds without significant differences were obtained.

There were 4,539,878 reads obtained from the *D. nobile* samples from four different altitudes after trimming the raw ITS2 sequencing data. Most of the length of these reads was distributed in the 200-400 bp region. The result of alpha analysis revealed no significant difference in the observed OTUs (Figure 1A) and Chao1 index (*p* = 0.031) (Figure 1B). Meanwhile, the Shannon index (*p* = 0.218) (Figure 1C) and Simpson index (*p* = 0.344) (Figure 1D) were not significantly different between each sample. Therefore, the community diversity of endophytes in *D. nobile* was not affected by different altitudes. Beta diversity was analyzed based on the weighted UniFrac distance. The PCA indicated that there were no significant differences in group diversity at different altitudes (Figure 1E). It means that under the habitat of Chishui city, the diversity of endophytes did not change significantly with changing altitudes, but whether the endophyte species would change cannot be shown.

On the other hand, a total of 818 metabolites of *D. nobile* were identified at different altitudes, including fatty acids, flavonoids and alkaloids. Meanwhile, metabolites could be found in each sample, which verified that metabolites were not determined by the altitude difference and that the expression levels of most metabolites were regulated by altitude. By one-way ANOVA (*p* < 0.05) (Figure 1F), 325 compounds with significant differences and 493 compounds with no significant difference were obtained.

### The species of endophytic fungi and the abundance of metabolites were regulated by altitude

**Figure 2:**
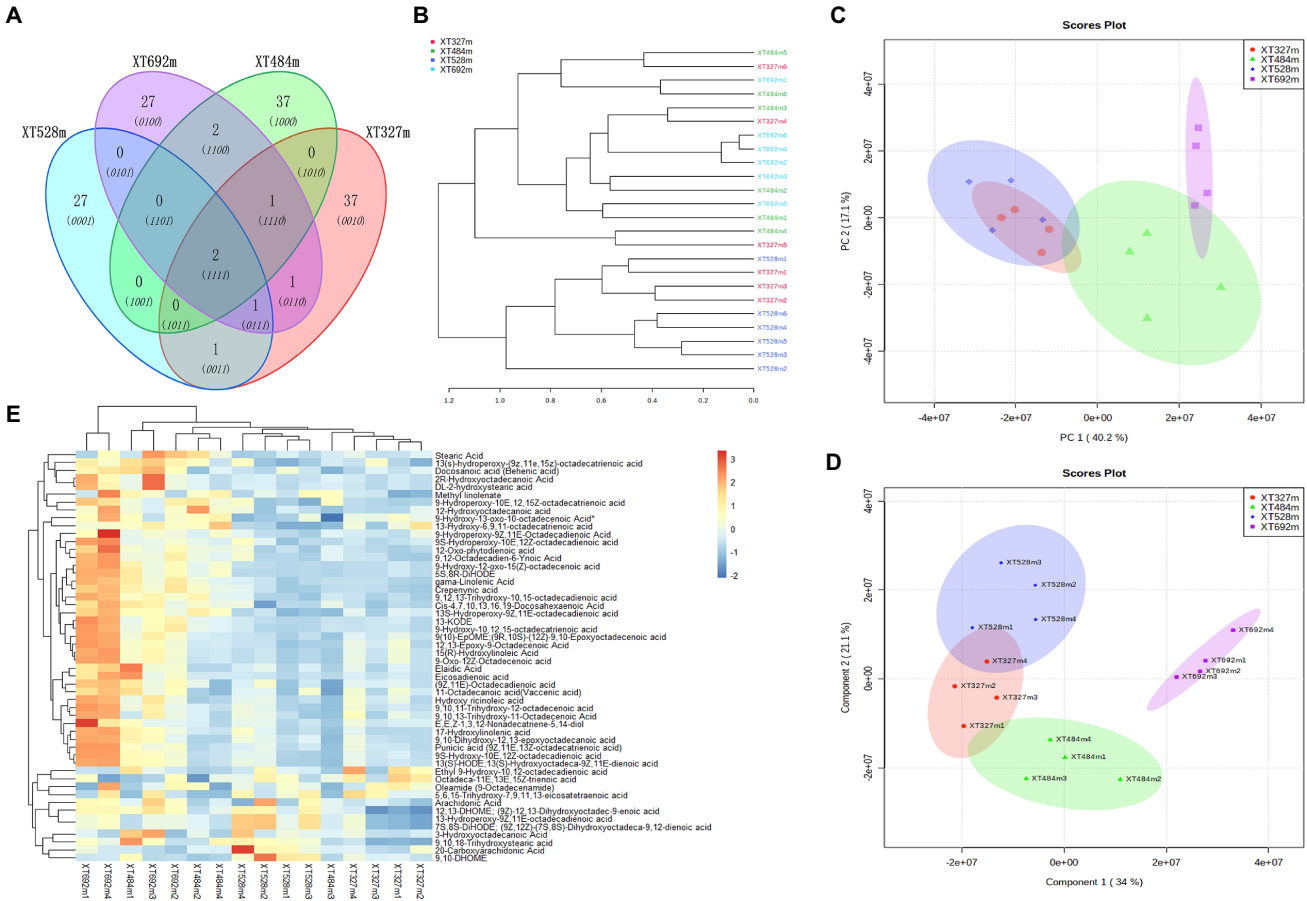
The species of endophytic fungi in *D. nobile* and the abundance of metabolites in *D. nobile* are different at different altitudes. **(A)** OTU Venn diagram of endophytic fungi in *D. nobile*. There were only two OTUs of endophytic fungi at four altitudes. **(B)** The development diagram of the Bray tree plot of the endophytes in the stems of *D. nobile*; **(C)** Score plots of PCA of metabolites of *D. nobile*; **(D)** Score plot of PLS-DA of metabolites at different altitudes, component 134%, component 2 21.1%, *R*^2^ = 0.89, *Q*^2^ = 0.74; **(E)** Heatmaps of metabolites of *D. nobile* at different altitudes.

The main medicinal components of medicinal plants are usually the primary or secondary metabolites of plants. The secondary metabolites of plants are an adaptation of plants to the environment, and their biosynthesis is the result of the interaction between plants and biotic and abiotic factors in the long-term evolution process. It plays an important role in the defense of adaptation to environmental stress, mutual competition and coevolution between plants, harm of plants to insects, herbivore feeding and invasion of pathogenic microorganisms. Therefore, compared with primary metabolism, the secondary metabolism of plants is more susceptible to external environmental conditions. Altitude, as a comprehensive ecological factor, forced a vital backdrop on the growth of plants, the infection of endophytic fungi, and the accumulation of metabolites. Through the identification of widely targeted metabolites, PCA showed that the contents of metabolites were clustered in different areas at different altitudes with a small part of the overlap (Figure 2C). The PLS-DA (Figure 2D) (Component 1 34% and Component 2 21.1%) showed the high reliability and good fit of the established model, with R^2^X = 0.892, R^2^Y = 0.963 and Q^2^ = 0.974. In the heatmap (Figure 2E), the top 50 variant metabolites screened out were observed to have different expression levels at different altitudes. The different metabolites were mainly concentrated in fatty acids, phenolic acids, alkaloids and flavonoids.

At the same time, the endophytic fungi in the stems of *D. nobile* at different altitudes were sequenced, it was found that the diversity of endophytes was not regulated by altitude and other factors; however, the endophyte species colonizing the plants were changed. The Wayne diagram (Figure 2A) showed that the endophytic fungi overlap OTUs under different conditions was very small; hence, most of them were different endophyte species, which was an interesting phenomenon that might be related to the environmental microbial communities at different altitudes. Additionally, the endophytic fungi in *D. nobile* at an altitude of 327 m were close to those at 484 m and 528 m, but the sample at 692 m was basically an independent branch. The above interest was shown in the development diagram of the Bray tree plot in the stems of *D. nobile* (Figure 2B). Altitude, as an abiotic stress, affects the colonization of microorganisms in plants and the accumulation of secondary metabolites, and there must be a certain correlation between them. The infection and colonization of endophytes are biological stresses of plants, and the feedback measures of plants to deal with biological stress include promoting the synthesis and accumulation of defensive metabolites (Yu et al., 2019). These defensive metabolites, such as flavonoids, phenols, alkaloids and fatty acids, are all responses to external biotic or abiotic stresses, thus regulating the relationship between plants and the environment (Isah, 2019; Erb and Kliebenstein, 2020). The biological stress caused by endophytes stimulates a series of signal transduction pathways in plant cells and acts on the synthesis pathway of plant metabolites, thus arousing plant defense. Therefore, plant-related microbial communities play an important role in improving plant growth, protecting against environmental stress (biotic and abiotic) and regulating the biosynthesis of secondary metabolites. Endophyte is a key factor in plant life (Pandey et al., 2022) and *D. nobile* is no exception. Obviously, the synthesis and accumulation of metabolites of *D. nobile* are closely related to the colonization of different endophytic fungi.

### *Toxicocladosporium rubrigenum* was hypothesized to be a typical endophytic fungus at high altitudes

**Figure 3:**
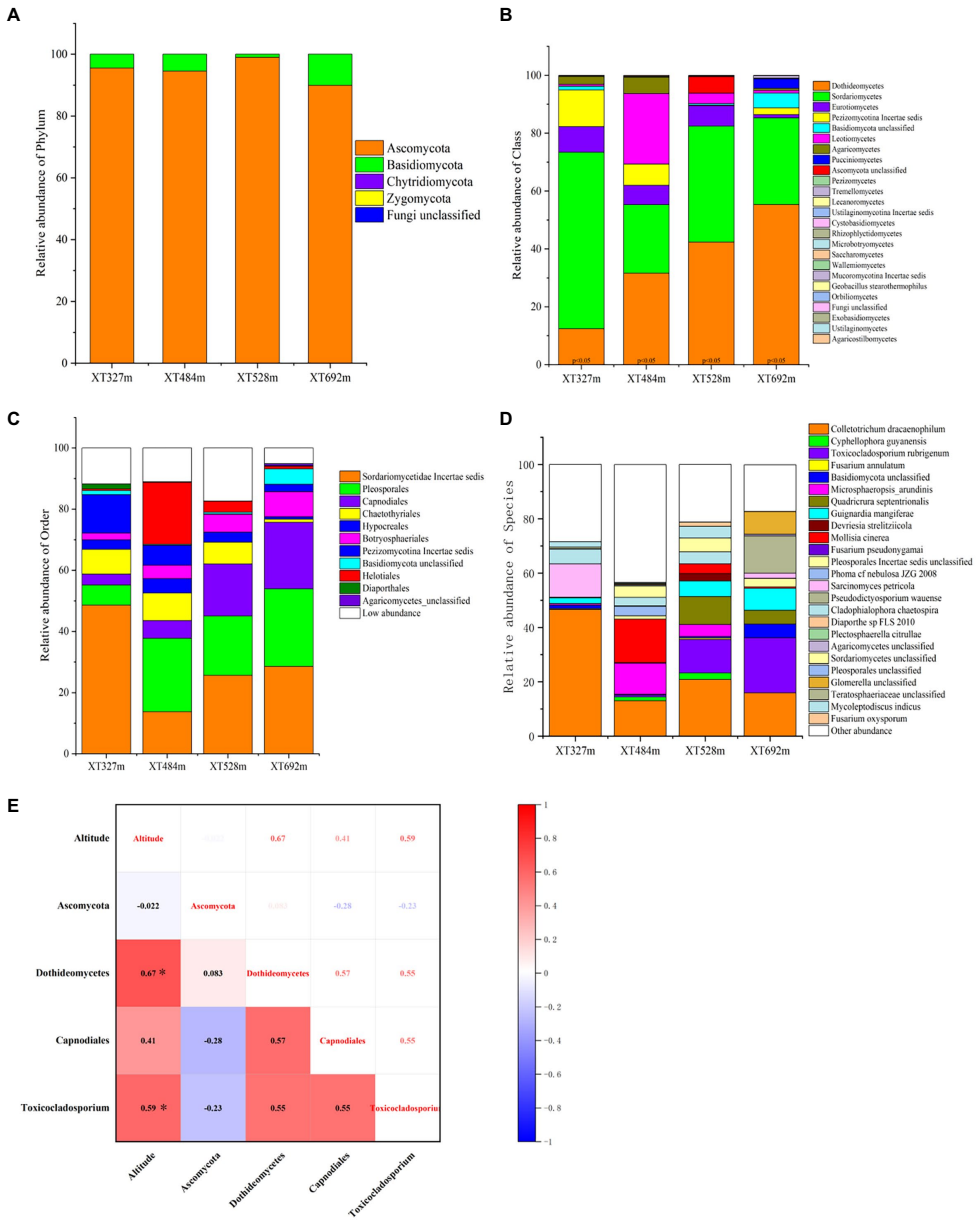
Endophyte relative abundance accumulation diagram of endophytic fungi in stems of *D. nobile* under different altitude conditions and the correlation diagram with altitude. **(A)** The relative abundance accumulation diagram at the phylum level; **(B)** at the class level; **(C)** at the order level; **(D)** at the species level; **(E)** the correlation diagram between the phyla, class, order, genera, species of *Toxicocladosporium rubrigenum* and the altitude based on the Spearman algorithm (*p < 0.05).

The community types of endophytic fungi in *D. nobile* will vary with their surroundings. At the phylum level, the maximum phyla in *D. nobile* were Ascomycota and Basidiomycota, among which Ascomycota was the absolute dominant phylum, and the abundance of Basidiomycota tended to increase with elevation (Figure 3A). The relative abundances of Dothideomycetes and Sordariomycetes were absolutely dominant at the class level (Figure 3B). At the low altitude of 327 m, Sordariomycetes accounted for 61.07% and Dothideomycetes accounted for 12.37%. With the increasing of the altitude, the relative abundance of Dothideomycetes gradually increased, reaching 55% at 692 m. The abundance of Sordariomycetes decreased with the altitude altering, among which 9 classes appeared in the 692 m habitat, and the microbial diversity at the class level reached the maximum (Figure 3B). The colonization of endophytes in *D. nobile* in response to altitude was well demonstrated here. The order level was analyzed next (Figure 3C). Pleosporales, Capnodiales, and Botryosphaeriales were supplemented following height; however, Sordariomycetae incertae sedis had the contrary regular pattern. In addition, Basidiomycota unclassified also had a notable response to altitude variation. At the species level (Figure 3D), the abundance of the *Colletotrichum dracaenophilum* strain decreased with elevation, which is the pathogen of plant anthracnose (Damm et al., 2019). The abundances of the *Cyphellophora guyanensis* strain and *Microsphaeropsis arundinis* were relatively high at middle altitudes (484 m and 528 m) but very low at high altitudes and low altitudes. The abundance of *Fusarium annulatum* was lowest in the XT692m sample. The abundance of the *Toxicocladosporium rubrigenum* strain and *Guignardia mangiferae* strain was higher at 692 m than at 372 m, which was the most meaningful. The taxonomic status of *Toxicocladosporium rubrigenum* belongs to Ascomycota; Pezizomycotina; Dothideomycetes; Capnodiales; Toxococladosporium (Crous et al., 2009). *Quadricrura septentrionalis* was significantly enriched in groups XT528m and XT692m. *Devriesia strelitziicola* and *Mollisia cinerea* were significantly different at 528 m. Nevertheless, *Phoma cf. nebulosa* JZG 2008 was significantly different at 484 m.

Based on Spearman, the correlation analysis between altitude and the abundance of Ascomycota, Dothideomycetes, Capnodiales, and *T. rubrigenum* (Figure 3E) showed that there was no close correlation with Ascomycota (*p* > 0.05). However, altitude was highly correlated with the abundance of Dothideomycetes, with a correlation coefficient of 0.67 (*p* < 0.001). Meanwhile, there was a correlation coefficient of 0.41 (*p* < 0.05) between altitude and Capnodiales. Finally, the correlation coefficient of height conditions and the strain *T. rubrigenum* was 0.59 (*p* < 0.01). All of the above were significant positive correlations, which lays great confidence in further discussing the relationship between endophytes and bionomic factors and plant metabolites.

### The content of fatty acid metabolites in *Dendrobium nobile* increased with height and some endophytes

**Figure 4:**
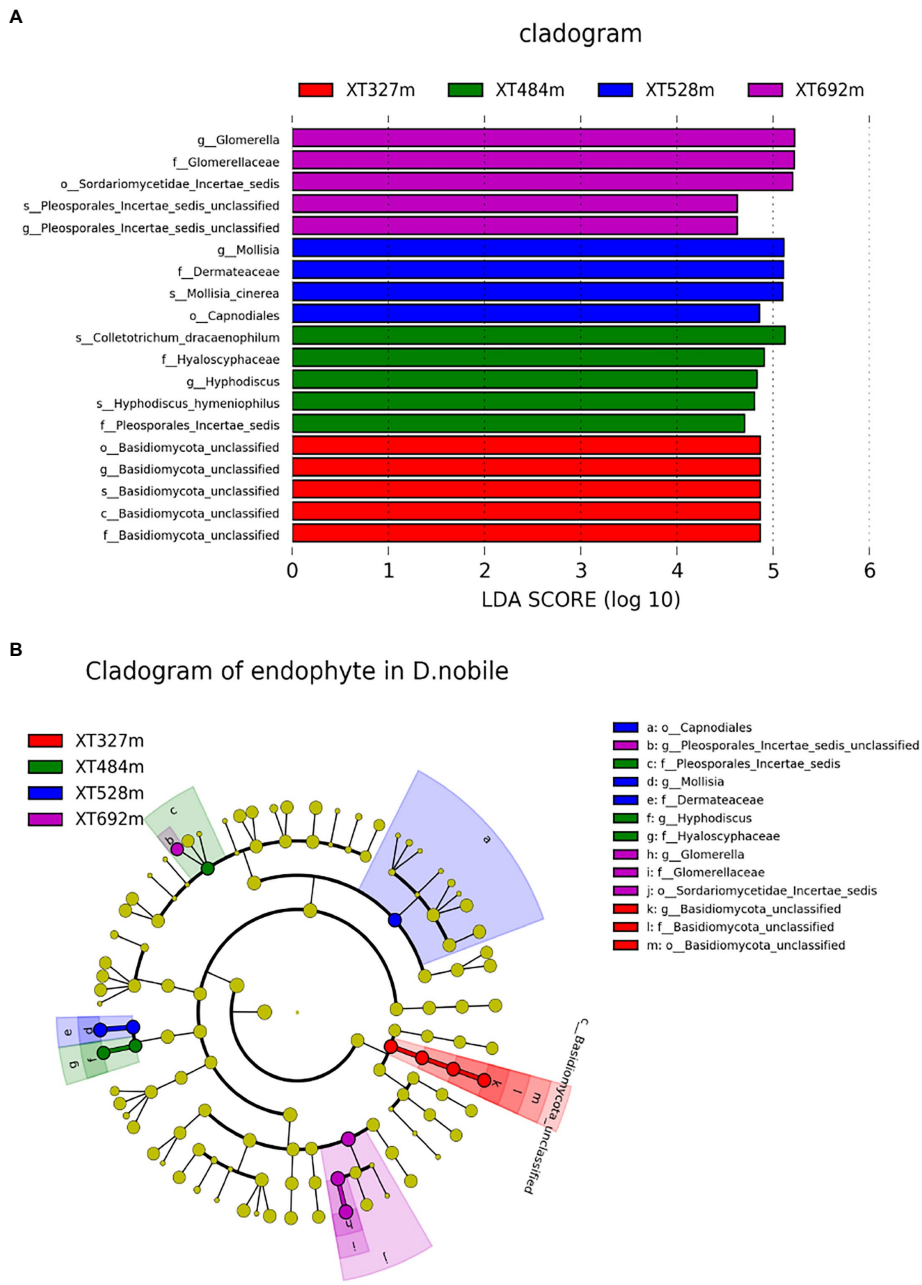
LEfSe analyses displayed taxa (from phylum to species) differences with absolute values of LDA > 2 for *D. nobile* at different altitudes. **(A)** Species with LDA scores greater than the set value of 2.0, that is, biomarkers with significant differences, and the length of the histogram represents the influence of significantly different species. **(B)** Phylogenetic diagram. Yellow shows the species with no significant difference, red shows the microbial groups that play an important role in XT327 m samples, green shows the microbial groups that play an important role in XT484 m, blue shows the microbial groups that play an important role in XT528 m, and purple shows the microbial groups that play an important role in XT692 m.

LEfSe analyses displayed taxa (from phylum to species) differences with absolute values of LDA > 2 for *D. nobile* at different heights (Figures 4A,B). The main endophytic fungi at 327 m were *Basidiomycota unclassified* from orders, families and genera, and the dominant endophytic fungi at 484 m were *Colletotrichum dracaenophilum*, Hyaloscyphaceae and Hyphodiscus, *Hyphodiscus hymeniophilusf* and *Pleosporales incertae sedis*. At an altitude of 528 m, the main endophytes are Capnodiales, Molisia, and Dermateaceae, *Mollisia cinerea* (species). And at 692 m, the main endophytes are *Pleosporales Incertae sedis* unclassified, *Sordariomycetae Incertae Sedis* Unclassified, Glomerellaceae and Glomerella. These biomarkers in each sample did not tend to change with increasing elevation; therefore, endophytes with typical representatives were considered in combination with endophytes that have the same trend as altitude, such as *T. ruberigenum*, *C. guyanensis* and *G. mangiferae*, *F. annulatum*, *M. arundinis*, *D. strelitziicola*, *F. pseudonygamai*, *Phoma cf. nebulosa* JZG 2008, *Q. septentrionalis*, *M. cinerea*, and *S. petricola*.

**Figure 5:**
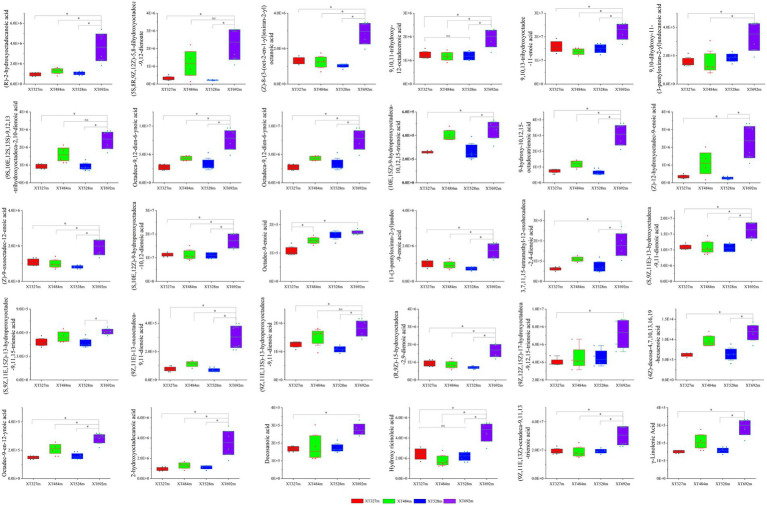
The content of identified fatty acid compounds in the stems of *D. nobile* at different altitudes. There were 30 fatty acid metabolites in *D. nobile* whose content increased with elevation (*p < 0.05, ns indicates no significant difference).

After screening, there were 30 kinds of fatty acid compounds whose content increased with increasing altitude (Figure 5; Supplementary Table S1). 9-hydroxy-10,12,15-octadecatrienoic acid, (5S,8R,9Z,12Z)-5,8-dihydroxyoctadeca-9,12-dienoate, (Z)-12-hydroxyoctadec-9-enoic acid, Hydroxy ricinoleic acid, 9,10,11-trihydroxy-12-octadecenoic acid, (9Z,11E,13S)-13-hydroperoxyoctadeca-9,11-dienoic acid, (9Z,11E)-13-oxooctadeca-9,11-dienoic acid; (10E,15Z)-9-hydroperoxyoctadeca-10,12,15-trienoic acid, etc., all of which are metabolites of octadecenoic acid in free fatty acids. Furthermore, octadecenoic acid is closely related to the signal molecules of plants. It has been reported that plants are infected under the external endophytic stress, some fatty acids act on plant hormone signaling molecules, such as jasmonic acid (JA) (Han et al., 2020), and then the expression of secondary metabolite-related transcription factors and synthesis genes is regulated, and the synthesis of secondary metabolites in plants is changed (Ho et al., 2020). Jasmine esters (JAs) are cyclopentanones derived from fatty acids belonging to the family of oxygenated fatty acid derivatives, collectively known as lipid oxides, which are produced by the oxidative metabolism of polyunsaturated fatty acids in plants (Yu et al., 2019). The intermediate metabolites involved in the synthesis pathway of octadecenoic acid, such as linolenic acid and 3,7,11,15-tetramethyl-12-oxohexadeca-2,4-dienoic acid, were detected in our data and showed the same variation tendency with elevation. It follows that altitude stress has a net expansionary effect on the accumulation of fatty acid metabolites in plants, especially octadecenoic acid components. Simultaneously, as the ecological niche changes, the abundance and species of the endophytic fungi, such as *Toxicocladosporium.*spp., *Sordariomycetidae Incertae sedis.spp*., *Glomerella.spp*. were tested and showed the same trend as fatty acids. It is speculated that the infection and colonization of endophytic fungi are positively correlated with fatty acid metabolites of octadecenoic acid, which is realized by regulating the signal molecular pathways of fatty acids in plants.

### The representative endophytes of *Dendrobium nobile* at high altitude are closely related to the upregulation of fatty acid metabolites

**Figure 6:**
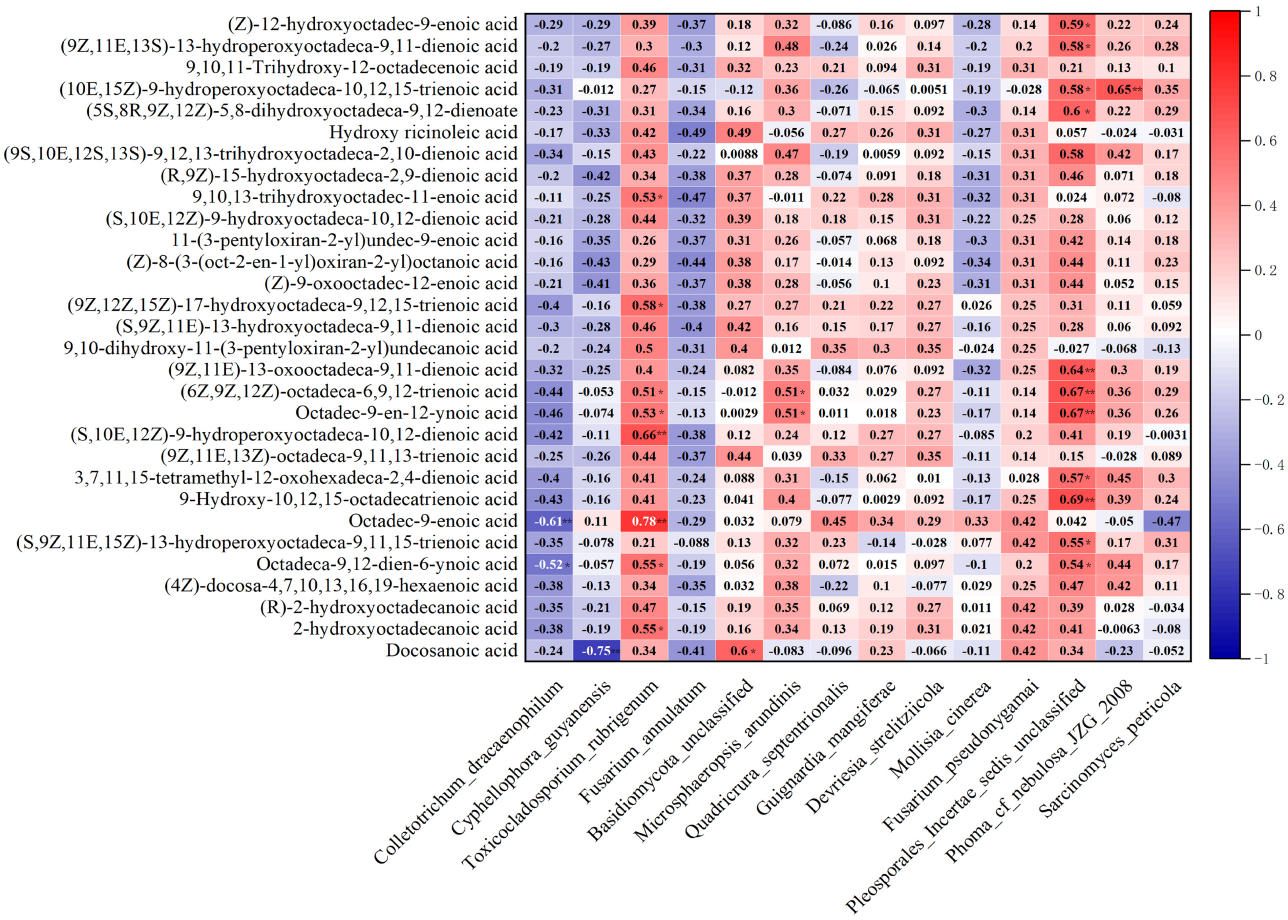
Correlation analysis between the dominant endophytes and dominant fatty acid metabolites of *D. nobile* at different altitudes. The red and blue indicate positive and negative correlations, and the color depth indicates strong correlation (**p < 0.01, *p < 0.05).

The correlation heatmap based on Spearman’s correlation showed that the key microbial populations were correlated with fatty acid metabolites (Figure 6). The vast majority of endophytes were positively correlated with every fatty acid metabolite, except for the four stains: *C. dracaenophilum*, *C. guyanensis*, *M. cinerea* and *F. pseudonygamai*, which were negatively correlated with the metabolites. In particular, the performance of *T. rubrigenum* and *Pleporales incertae sedis unclassified* is particularly outstanding. The correlation coefficient between *T. ruberigenum* and Octadec-9-enoic acid (12,13-DHOME) was 0.78 (*p* < 0.01) and that between *T. ruberigenum* and (S,10E,12Z)-9-hydroperoxyoctadeca-10,12-dienoic acid was 0.66 (*p* < 0.01). On its heels, the correlation coefficients of (9Z,12Z,15Z)-17-hydroxyoctadeca-9,12,15-trienoic acid and 9,10,13-trihydroxyoctadec-11-enoic acid with *T. rubrigenum* were 0.58 (*p* < 0.05) and 0.53 (*p* < 0.05), respectively. The correlation coefficient between *Pleporales incertae sedis unclassified* and 9-hydroxy-10, 12, 15-octadecatrienoic acid was 0.69 (*p* < 0.01), 0.64 (*p* < 0.01) with (9Z,11E)-13-oxooctadeca-9,11-dienoic acid, and 0.67 (*p* < 0.01) with (6Z,9Z,12Z)-octadeca-6,9,12-trienoic acid (γ-Linolenic Acid) and crepenynic acid. Next, the coefficient of correlation of this fungus and 9-hydroxy-12-oxo-15 (z)-octadecadienoic acid was 0.59 (*p* < 0.05). The number 0.58 (*p* < 0.05) appeared twice among (9Z,11E,13S)-13-hydroperoxyoctadeca-9,11-dienoic acid, (10E,15Z)-9-hydroperoxyoctadeca-10,12,15-trienoic acid and *Pleporales incertae sedis unclassified*. In addition, the interdependency (0.65, *p* < 0.01) between *Phoma cf. nebulosa* JZG 2008 and (10E,15Z)-9-hydroperoxyoctadeca-10,12,15-trienoic acid, even pertinence (0.6, *p* < 0.05) with Basidiomycota unclassified and Docosanoic acid (Behenic acid), was calculated in this paper. All the observed data showed that only *M. arundinis* was closely related to (6Z,9Z,12Z)-octadeca-6,9,12-trienoic acid and Octadec-9-en-12-ynoic acid (0.51, *p* < 0.05). None of the other endophytic fungi showed a significant correlation with the metabolites. Based on the above data, the following conclusions were speculated: the fatty acid metabolites of *D. nobile* were accelerated by the colonization of strain *T. ruberigenum*, *P. incertae sedis*, *Phoma cf. nebulosa* JZG2008, *Basidiomycota* spp. and *M. arundinis*. In contrast, strains that have negative associations with metabolites are not our favorite microbes, among which *C. dracaenophilum* was significantly correlated with Octadec-9-enoic acid (−0.61, *p* < 0.05), *and C. guyanensis* was also significantly correlated with Docosanoic acid (−0.75, *p* < 0.01). The other negative correlation coefficients (*p* > 0.05) were not statistically significant. Therefore, colonization by some endophytic fungi dependent on altitude were beneficial to the synthesis and accumulation of fatty acid metabolites in *D. nobile*. It was also confirmed in related reports that the infection of the endophyte *Fusarium verticillioides* in *Zea mays* resulted in a change in plant lipid (fatty acids, oxylipins, and sphingolipids) metabolism, and C16:1, C18:0, and C18:2 significantly increased in the fourth generation (Beccaccioli et al., 2021).

## Discussion

### Triangle relationship among ecological factors, endophytes, and host metabolites

At high altitudes, more significant anti-fatigue-related pharmacological effects were shown in medicinal plants through natural selection and habitat coevolution (Zhu et al., 2022). For the plants *Horedum vulgare, Triticum aestivum* and *Pisum sativum*, with increasing altitude, the respiration of these plants would be enhanced, which might be advantageous to the higher metabolic activities of these plants (Kumar et al., 2007). It is also represented in other plants and the content of flavonoids in *Ginkgo biloba* increased by more than 150% with elevation (530–2,310 m) (Fu et al., 2022). Hence, the medicinal composition of numerous medicinal plants is significantly regulated by changes in ecological factors. Moreover, it is almost common sense that healthy plants are colonized by diverse microorganisms, regulating the physiology and development of host. Then, are these colonizing microbes responsive to environmental factors? There were evidences that the endophytes Zygomycota, Cercozoa, Glomeromycota, Chymomycota, and Rozellomycota in *Stipa purpurea* posed strongly positive associations with altitude (Liu et al., 2022). At the same time, along with elevation, the endophyte network in *Ginkgo biloba* leaves became more complex, which may be one of the strategies of endophytes in leaves to respond to ecological factors and one means to cope with the increase in flavonoids (Fu et al., 2022). After all, flavonoids increase in addition to altitude. How is such an obvious triangular relationship among ecological factors, endophytes and host metabolites realized? Once the fungus reaches the plant root, it penetrates and grows intercellularly in the cortex through a functionally distinct invasive growth (IG) MAPK cascade (invasive growth (IG) MAPK cascade) with different functions. For example, *Fusarium oxysporum* invades plants and secretes a series of effector proteins, and RALF (rapid alkalization factor) effectively induces host alkalization, thus stimulating MAPK-driven invasive growth and promoting strain colonization in the host (Palmieri et al., 2020). Then, the protein factors secreted by endophytes are absorbed by plant cells and transmitted to neighboring cells, and the metabolic state of plant cells is changed by metabolic activation to regulate the synthesis and accumulation of metabolites (Djamei et al., 2011). Therefore, altitude can regulate plant metabolites by regulating the invasion of microorganisms. The replanting experiment of a single fungus isolated from sterile Brassica plants also revealed various influences of endophytic fungi on plant performance, including the continuous transformation process from parasitism to symbiosis. More importantly, the effects of interactions on plant health can be readjusted through host genetics, host nutrition and local environmental conditions (Mesny et al., 2021). It has been revealed that there is a cross-talk interaction triangle relationship between external environmental factors, plant endophytes and plant metabolites.

### Ailtitude and endophytes

Actually, elevational gradients are an ideal system to study how specialization between plant hosts and microbial symbionts can covary with changes in environmental conditions, as the same host species can be found along the entire gradient, while discovering large changes in abiotic conditions (Cobian et al., 2019). In an archipelago-wide survey of >100 host plants at 2300 m, many environmental factors, mainly elevation and cell-level evapotranspiration associated with endophyte community composition, were found (Darcy et al., 2020). The foliar endophyte communities were structured by elevation and aspect in *Metrosideros polymorpha* (Zimmerman and Vitousek, 2012). In this study, the diversity of endophytes of *D. nobile* did not significantly change with elevation (327 m, 484 m, 528 m, 692 m), but the distribution of endophytic flora was significantly different in each sample. The main groups of endophytes of *D. nobile* planted at the highest mountain (692 m) were *Sordariomycetidae Incertae sedis*, *Soriomycetidae incertaesedis unclassified*, Glomerella and Glomerella. At low position (327 m), the main endophytic fungi are Basidiomycota. The endophyte communities with gradient changes in elevation were considered to be the most interesting, including *T. rubrigenum, G. mangiferae, F. annulatum*, *M. arundinis*, *D. strelitziicola*, *F. pseudonygamai*, *Phoma cf. nebulosa* JZG 2008, *Q. septentrionalis*, *M. cinerea* and *S. petricola*. Accordingly, the endophyte species in *D. nobile* is also regulated by altitude.

### Ailtitude and metabolites

The metabolites of *D. nobile* were detected by UPLC–ESI–MS/MS analysis and the fatty acid metabolites in *D. nobile* changed synergistically with increasing altitude, especially long-chain fatty acids with 18 carbons. Therefore, an analysis focusing on the relationship between fatty acid metabolites and endophytes will be more convincing. Fatty acids and lipids are the main and essential components of all plant cells, which not only provide structural integrity and energy for various metabolic processes but also act as intracellular and extracellular signal transduction media. Palmitic (16: 0), stearic (18, 0), oleic, linoleic and linolenic acids are the most common fatty acid metabolites in plant lipids, among which the levels of the unsaturated FAs (those hat carry double bonds between carbons) 18:1, 18:2, and 18:3 are particularly important in plant defense (Lim et al., 2017). The C18 fatty acid metabolites of *D. nobile* significantly changed with altitude and the types of endophytes. Although the content of alkaloids in *D. nobile* is a quality marker, other metabolites also play an important role in pharmacological activity and quality control (Nie et al., 2020). Metabolites of fatty acids provide the material basis for the synthesis of sesquiterpene alkaloids and they are also signal transmission molecules to address external pressures. JA, an oxidized lipid, plays a crucial role in both biotic and abiotic stresses in plants, which is initiated by linolenic acid and passed through lipoxygenase (LOX), Allene oxide synthase (AOS) and Allene oxide cyclase (AOC), leading to the production of 12-oxo-phytodionoic acid (12-OPDA) and then through a series of oxidations to form JA. In *D. nobile*, the content of γ-linolenic acid ((6Z,9Z,12Z)-octadeca-6,9,12-trienoic acid) at an altitude of 692 m which was significantly higher than that in plants at 372 m and 528 m. Under catalysis by the lipoxygenase (LOX) enzyme, 13-hydroperoxy-octadecatrienoic acid ((S)-HPOD) was synthesized. The compounds 13 (S)-hydroperoxy-(9z, 11e, 15z)-octadecatrienoic acid (3 (S)-HPODE) and 13 s-hydroperoxy-9z, 11e-octadecadienoic acid were detected, all of which have a tendency to increase with altitude. Under the continuous action of allene oxide synthase (AOS), 11-(3-pentyloxiran-2-yl) undec-9-enoic acid was obtained, whose content in the 692 m samples of *D. nobile* was significantly higher than that in the other samples (*p* < 0.05). 3,7,11,15-tetramethyl-12-oxohexadeca-2,4-dienoic acid (12-Oxo-phytodienoic acid, PDA) was synthesized by allene oxide cyclase (AOC) as a catalyst, and it was also altitude dependent. The content of 12-oxo-phytodienoic acid in the 692 m samples reached the maximum, which was significantly higher than that in the 327 m and 528 m samples. Thus, the synthesis pathway of the plant signal molecule JA is positively regulated by altitude, which is similar to the mechanism of JA synthesis related to the stress and pressure felt by plants (Ghorbel et al., 2021).

### Endophytes and metabolites

Endophytic fungi live in healthy plant tissues and organs at one or all stages of their life cycles, and their distributions and population structures are believed to be drastically affected by host genetics and environmental conditions. Endophytes can evolve (Chen et al., 2019) together with host plants for a long time, which can not only promote plant growth but also continuously promote the accumulation of active components of medicinal plants and even produce the same physiological active substance (Sharma et al., 2021), improving the quality and yield of medicinal plants. Endophytes were positively regulated by altitude and were related to fatty acid metabolism, including *T. rubrigenum*, *P. incertae sedis unclassified*, *Phoma cf. nebulosa* JZG 2008, and *Basidiomycota* sp. in *D. nobile*. *T. rubrigenum* isolated from *Eucalyptus* spp. was first reported in 2009 (Crous et al., 2009). Then, the microorganism of the genus *Toxococladosporium* was gradually reported and it was found that the endophyte of the genus *Toxococladosporium* could be isolated from *Pilosocereus gounelei* subsp. *Gounelei* and *Melocactus zehntneri* (Bezerra et al., 2017; Mounes Bakhshi, 2022). Through correlation analysis, it can be seen that the above three dominant endophytes have a positive correlation with fatty acid metabolites of *D. nobile* at different altitudes; however, *C. dracaenophilum*, *C. guyanensis* and *M. cinerea* have a negative correlation with metabolites, which shows that not all endophytes symbiotic with the host plant are beneficial to the plant itself without showing symptoms. Instead, endophytes and plants have formed a certain regulatory network through long-term development and evolution, thus maintaining the steady state between plants and endophytes.

## Conclusion

Endophytic ITS sequencing and identification of widely targeted metabolites of four wild-grown *D. nobile* samples were tested and analyzed. Elevation is not a major determinant of *D. nobile* endophytic fungal diversity but regulates endophyte species. High altitude (692 m) was propitious to the infection of biomarker endophytic fungi and significantly upregulated the synthesis and accumulation of fatty acid metabolites, especially intermediate metabolites in the JA synthesis pathway. A significant positive correlation was shown between the fatty acid metabolites and altitude-representative endophytes through correlation analysis. It was speculated that in *D. nobile*, the endophyte community stimulates the synthesis of plant hormones to regulate plant growth and metabolism.

## Data availability statement

The raw data are deposited in the NCBI repository, BioProject ID: PRJNA948714.

## Author contributions

YH: conceptualization, supervision, project administration, and funding acquisition. DT and QF: data curation. QF: formal analysis. DT, XW, and CB: resources. DW, CB, and JX: methodology. LQ, XW, and JY: software. DW: Validation. YZ: writing – original draft. JX: writing – review and editing. All authors contributed to the article and approved the submitted version.

## Funding

This research was funded by Guizhou Engineering Research Center of Industrial Key-technology for Dendrobium Nobile (QJJ[2022]048). Guizhou Provincial Education Department (QJJ[2022]006). The Department of Science and Technology of Guizhou Province (QKHZC[2020]4Y072, QKHZC[2021]420). Science and Technology Plan of Zunyi (ZSKHHZ [2020] 321).

## Conflict of interest

CB was employed by Guangxi Shenli Pharmaceutical Co., Ltd; JY was employed by Chishui Xintian Chinese Medicine Industry Development Co., Ltd.

The remaining authors declare that the research was conducted in the absence of any commercial or financial relationships that could be construed as a potential conflict of interest.

## Publisher’s note

All claims expressed in this article are solely those of the authors and do not necessarily represent those of their affiliated organizations, or those of the publisher, the editors and the reviewers. Any product that may be evaluated in this article, or claim that may be made by its manufacturer, is not guaranteed or endorsed by the publisher.

## Supplementary material

The Supplementary material for this article can be found online at: https://www.frontiersin.org/articles/10.3389/fmicb.2023.1128956/full#supplementary-material

## Footnotes

^1^ http://www.anpel.com.cn/

^2^ https://www.shimadzu.com.cn/

^3^ https://www.thermofisher.cn/cn/zh/home/brands/applied-biosystems.html

^4^ https://huttenhower.sph.harvard.edu/galaxy/

^5^ https://www.metaboanalyst.ca
